# The impact of freeze-drying infant fecal samples on measures of their bacterial community profiles and milk-derived oligosaccharide content

**DOI:** 10.7717/peerj.1612

**Published:** 2016-01-21

**Authors:** Zachery T. Lewis, Jasmine C.C. Davis, Jennifer T. Smilowitz, J. Bruce German, Carlito B. Lebrilla, David A. Mills

**Affiliations:** 1 Department of Food Science and Technology, University of California, Davis, CA, United States; 2 Foods For Health Institute, University of California, Davis, CA, United States; 3 Department of Chemistry, University of California, Davis, CA, United States; 4 Department of Viticulture and Enology, University of California, Davis, CA, United States

**Keywords:** Freeze-drying, Lyophilization, Human microbiome, Fecal microbiome, Human milk oligosaccharides, Infants

## Abstract

Infant fecal samples are commonly studied to investigate the impacts of breastfeeding on the development of the microbiota and subsequent health effects. Comparisons of infants living in different geographic regions and environmental contexts are needed to aid our understanding of evolutionarily-selected milk adaptations. However, the preservation of fecal samples from individuals in remote locales until they can be processed can be a challenge. Freeze-drying (lyophilization) offers a cost-effective way to preserve some biological samples for transport and analysis at a later date. Currently, it is unknown what, if any, biases are introduced into various analyses by the freeze-drying process. Here, we investigated how freeze-drying affected analysis of two relevant and intertwined aspects of infant fecal samples, marker gene amplicon sequencing of the bacterial community and the fecal oligosaccharide profile (undigested human milk oligosaccharides). No differences were discovered between the fecal oligosaccharide profiles of wet and freeze-dried samples. The marker gene sequencing data showed an increase in proportional representation of *Bacteriodes* and a decrease in detection of bifidobacteria and members of class Bacilli after freeze-drying. This sample treatment bias may possibly be related to the cell morphology of these different taxa (Gram status). However, these effects did not overwhelm the natural variation among individuals, as the community data still strongly grouped by subject and not by freeze-drying status. We also found that compensating for sample concentration during freeze-drying, while not necessary, was also not detrimental. Freeze-drying may therefore be an acceptable method of sample preservation and mass reduction for some studies of microbial ecology and milk glycan analysis.

## Introduction

Infants go from the womb to abruptly encountering the full microbial diversity of their new ex-utero environment. Early microbial colonization impacts the long-term adult microbial ecosystem (Biasucci et al., 2008), and is likely a critical ecological window that influences health trajectory throughout life (Blaser & Falkow, 2009; Dominguez-bello et al., 2011; Scholtens et al., 2012; Cho et al., 2012). Infant fecal samples are commonly studied to investigate the impacts of factors such as breastfeeding on the development of the gut microbiota and subsequent health effects. As some of the effects of breastfeeding on the microbiota are mediated by selective microbial consumption of the oligosaccharides found in mother’s milk, the milk-derived fecal oligosaccharide profile is of scientific and practical interest (Lewis et al., 2015). Human milk oligosaccharides have also been shown to have antiadhesive properties, to modulate immune cell responses, and are of increasing interest as therapeutic agents in the diet (Bode, 2012; Smilowitz et al., 2014).

Comparisons of published data sets from infants around the world show differences between the gut microbiomes of infants from different countries (Jost et al., 2012; Yatsunenko et al., 2012; Avershina et al., 2013; Roos et al., 2013; Azad et al., 2013; Abrahamsson et al., 2013; Huda et al., 2014; Lewis et al., 2015). The study of infants living in disparate environmental contexts will be necessary to our understanding of evolutionarily-selected milk adaptations. However, the preservation of fecal samples from infants in remote locations until lab processing can be a challenge given the limitations of the infrastructure in many potentially interesting locations. Freeze-drying (also known as lyophilization) is a common method of preserving material by removing water in a low-pressure, low temperature environment whereby water directly sublimates. It is an important technique in a variety of different industries (i.e., pharmaceutical and food), and is useful in some scientific fields for biological sample preservation in remote areas where appropriate refrigeration prior to lab analysis is impossible or cost prohibitive. Freeze-drying stabilizes samples and reduces the weight, reducing the risk and cost of shipping samples over large time periods and/or distances. Freeze-drying infant fecal samples may open up new geographic areas for investigation and improve the data from existing field studies. However before researchers can use freeze-dried samples to study microbial communities, their determinants (e.g., human milk oligosaccharides), and their downstream byproducts the effects of lyophilization on these factors must first be understood.

The impact of freeze-drying on the extraction of oligosaccharides from samples is unknown. Several methods have been developed for oligosaccharide extraction for both wet and freeze-dried fecal samples, using analytical techniques such as high-performance anion-exchange chromatography, colorimetric methods, and gas chromatography/mass spectrometry (Sabharwal et al., 1984; Sabharwal, Sjoblad & Lundblad, 1991; Moro et al., 2005). These methods have been successful in extracting oligosaccharides from wet and lyophilized feces, but to our knowledge there has not been a study showing whether freeze-drying fecal samples affects the integrity of the sample and analyses.

Conversely, DNA-based studies are obvious candidates for the use of freeze-drying on field samples, as DNA has been shown to be relatively stable in a variety of freeze-dried preparations (Gianaroli et al., 2012; Van der Heijden, Beijnen & Nuijen, 2013). Studies on macro-organisms have yielded a spectrum of conclusions on freeze-drying field samples for DNA extraction and further study ranging from mixed to positive (Wasser et al., 1997; Straube & Juen, 2013). The few studies available on microorganisms, however, have shown advantages to using freeze-dried samples which may include better DNA yield from extraction, at least from fecal samples (Ruiz & Rubio, 2009; Rapp et al., 2010). Freeze-drying may protect microbial DNA in fecal samples from hydrolytic damage and enzymatic degradation (Machiels et al., 2000). The effects of freeze drying on measures of microbial ecology, however, are poorly understood, especially in relation to current marker-gene amplicon sequencing methods. Numerous studies have shown that factors ranging from primer choice, DNA extraction method, sample preservation method, and kit contamination influence the output of marker gene sequencing-based microbial ecology studies to various degrees (Maukonen, Simões & Saarela, 2012; Ghyselinck et al., 2013; Dominianni et al., 2014; Wesolowska-Andersen et al., 2014; Weiss et al., 2014; Mennerat & Sheldon, 2014; Rubin et al., 2014; Albertsen et al., 2015; Voigt et al., 2015; Wagner-Mackenzie, Waite & Taylor, 2015; Walker et al., 2015). If freeze drying has differential effects on the DNA extraction efficiency of different types of bacteria (due to cell wall composition, the presence of an exopolysaccharide capsule, biofilm formation, sporulation, or any other reason), it would bias the relative abundance data output of DNA-based studies. Here we investigated these potential confounding factors on fecal oligosaccharide and microbiota analysis using a test set of infant fecal samples.

## Materials and Methods

### Sample collection

#### Infant stool samples

Twenty-four prospective mothers were enrolled in the Foods for Health Institute Lactation Study at UC Davis at approximately 34 weeks of gestation and asked to fill out detailed questionnaires which included information about their infant’s diet throughout the study. Infant fecal samples were collected at 340–400 days of life from twenty-four breast-fed term infants born to women in the study. Parents transferred their infant fecal samples into sterile plastic tubes and were instructed to immediately store the samples in −20°C until transported by study personnel. Fecal samples were transported to the laboratory on ice packs and stored at −80°C before processing. The UC Davis Institutional Review Board approved all aspects of the study (approval #216198) and written informed consent was obtained from all subjects. This trial was registered on clinicaltrials.gov (ClinicalTrials.gov Identifier: NCT01817127).

### Freeze drying and DNA extraction

Each fecal sample was split into two analysis streams. A portion of each sample (approximately 1.5 g) was taken and freeze-dried using a Labconco FreeZone 4.5 freeze-dry system until dry. Each sample was weighed before and after freeze-drying, and the percent mass loss was calculated for each sample. Each freeze-dried sample was sub-divided into two arms for DNA extraction, one in which the amount of mass loss incurred during freeze drying was accounted for before DNA extraction (“Low mass”), and one in which the DNA extraction kit (ZR Fecal DNA MiniPrep Kit; Zymo Research, Irvine, CA, USA) manufacturer’s instructions (150 mg of sample) were followed without accounting for the effects of freeze drying (“High mass”). These two conditions (High and Low mass), along with a non-lyophilized aliquot from each fecal sample were used for DNA extraction. This included a bead-beating step using a FastPrep-24 Instrument (MP Biomedicals, Santa Ana, CA, USA) for 2 min at 25°C at a speed of 6.5 m/s. In a few cases, the default amount of lysis solution (750 µl) was insufficient to reconstitute the freeze-dried samples, and the addition of more (up to the capacity of the tube) was necessary to fully rehydrate the samples. One sample (#16) did not have sufficient feces to perform all the analysis, and therefore no “Low mass” condition was tested. All DNA extractions were performed in duplicate.

### Sequencing and analysis

#### Illumina sequencing—V4 region

Duplicate DNA extractions for each of the 24 samples under each condition (High mass, Low mass, and Wet) were prepared for marker gene sequencing as previously described (Caporaso et al., 2011) with the following modifications. Universal barcoded primers with Illumina sequencing adapters (adapters are italicized and an example barcode is highlighted in bold) V4F (5′-*AATGATACGGCGACCACCGAGATCTACACTCTTTCCCTACA CGACGCTCTTCCGATCT* **ACTGCTGA**GTGTGCCAGCMGCCGCGGTAA-3′) and V4Rev (5′-*CAAGCAGAAGACGGCATACGAGATCGGTCTCGGCATTCCTGCT GAACCGCTCTTCCGATCT*CCGGACTACHVGGGTWTCTAAT-3′) were used to PCR amplify the V4 region of the 16S rRNA gene (Caporaso et al., 2011). PCR reactions contained 7.5 µl 2x GoTaq Green Master Mix (Promega, Madison, WI, USA), 0.6 µl 25 mM MgCl_2_, 3.6 µl water, 1.5 µl forward and 0.3 µl reverse primers (0.2 µM final concentration), and 1.5 µl DNA. A negative control was also included into which water was added in the place of DNA. A portion of each reaction was electrophoresed in a 0.8% agarose gel and stained with GelGreen (Phenix, Candler, NC, USA). The DNA band for each sample was visually categorized by brightness and size for quality control. All samples were pooled (5 µl of each reaction for samples with bright bands, 10 µl for faint samples with bands, and 12 µl for samples with non visible bands) and purified with the QIAquick PCR Purification Kit (QIAGEN, Valencia, CA, USA) according to the manufacturer’s instructions. The pooled, purified amplicons were sequenced at the University of California-Davis DNA Technologies Core Facility on an Illumina MiSeq sequencing platform.

#### Sequence analysis

Illumina V4 16S rRNA gene sequences were demultiplexed and quality filtered using the QIIME 1.8 software package with default settings unless otherwise specified (Caporaso et al., 2010a). Reads were truncated after a maximum number of 3 consecutive low quality scores. The minimum number of consecutive high quality base calls to include a read (per single end read) as a fraction of the input read length was 0.75. The minimum acceptable Phred quality score was set at 20. Similar sequences were clustered into operational taxonomic units (OTUs) using open reference OTU picking with UCLUST software (Edgar, 2010). Taxonomy was assigned to each OTU with the Ribosomal Database Project (RDP) classifier (Wang et al., 2007) and the RDP taxonomic nomenclature (Cole et al., 2009). OTU representatives were aligned against the Greengenes core set (DeSantis et al., 2006) with PyNAST software (Caporaso et al., 2010b). PCoA (Principle Coordinate Analysis) plots were generated using the default beta diversity analysis parameters based off of a weighted UniFrac distance matrix (Lozupone & Knight, 2005). The sequencing data is available in the European Nucleotide Archive under study number ERP012928.

### Oligosaccharide analysis

#### Oligosaccharide extraction

Free oligosaccharides were extracted from aliquots of both the freeze-dried and non-lyophilized samples from 23 of the infants (sample for infant 18 did not have enough feces for both analyses) following previously reported methods for human milk oligosaccharide extraction from breast milk, with extra initial homogenization and solid phase extraction steps (Ninonuevo et al., 2006; Wu et al., 2010; Wu et al., 2011). A total of 20 mg of each of the samples were diluted with 200 µL water and shaken overnight. After centrifugation, 25 µL of supernatant was aliquotted onto a 96-well plate, followed by protein removal via ethanol precipitation. The resulting glycans were reduced with 1.0 M NaBH_4_ at 65°C in an incubator for 1.5 h. After reduction the samples were cleaned on solid phase extraction C8 cartridges, in which the eluent was collected along with a water wash. The flow-through was then purified on graphitized carbon cartridges by desalting with deionized water and eluted first with 20% acetonitrile in water, then 40% acetonitrile in 0.05% trifluoroacetic acid (v/v). The eluent fractions were collected in the same wells and the solvent was evaporated. The samples were reconstituted and diluted to an appropriate concentration for analysis.

#### Oligosaccharide analysis

The extracted oligosaccharides were analyzed on a nano-high performance liquid chromatography (HPLC)-Chip/time-of-flight (TOF) mass spectrometry system. The Agilent 1200 series HPLC system uses a capillary pump for sample loading and a nano pump for separation, all done on a microfluidic chip. The chip has a 40 nL enrichment column and a 75 µL × 43 mm analytical column packed with porous graphitized carbon. The samples are loaded by the capillary pump at a rate of 4.0 µL/min and a 2 µL injection volume onto the enrichment column. Chromatographic separation is accomplished with a binary gradient of aqueous solvent (3% acetonitrile:water (v/v) in 0.1% formic acid) and organic solvent (90% acetonitrile:water (v/v) in 0.1% formic acid). This system is coupled to an Agilent 6220 series TOF mass spectrometer via chip-cube interface. The instrument was calibrated by a dual nebulizer electrospray source with internal calibrant ions ranging from *m*∕*z* 118.086 to 2721.895. Data was collected in the positive mode following the method developed and optimized for oligosaccharide separation by Wu et al. (2010) and Wu et al. (2011).

Data was collected and processed using Agilent MassHunter Qualitative Analysis software, version B.03.01. Oligosaccharide compounds were identified with the *Find by Molecular Feature* function with a 20 ppm mass error parameter when compared to theoretically calculated masses based on previously developed libraries and possible protein-linked glycans (Wu et al., 2010; Wu et al., 2011; Nwosu et al., 2012). The oligosaccharides were divided into four glycan classes: fucosylated (any structure with fucose), sialylated (any structure with sialic acid), fucosylated and sialylated, and non-fucosylated neutral. Relative class abundance was calculated by dividing each class abundance by the total oligosaccharide amount for each infant. Paired *t*-tests were used to determine if there were differences between glycan content of freeze-dried and wet feces.

**Figure 1:**
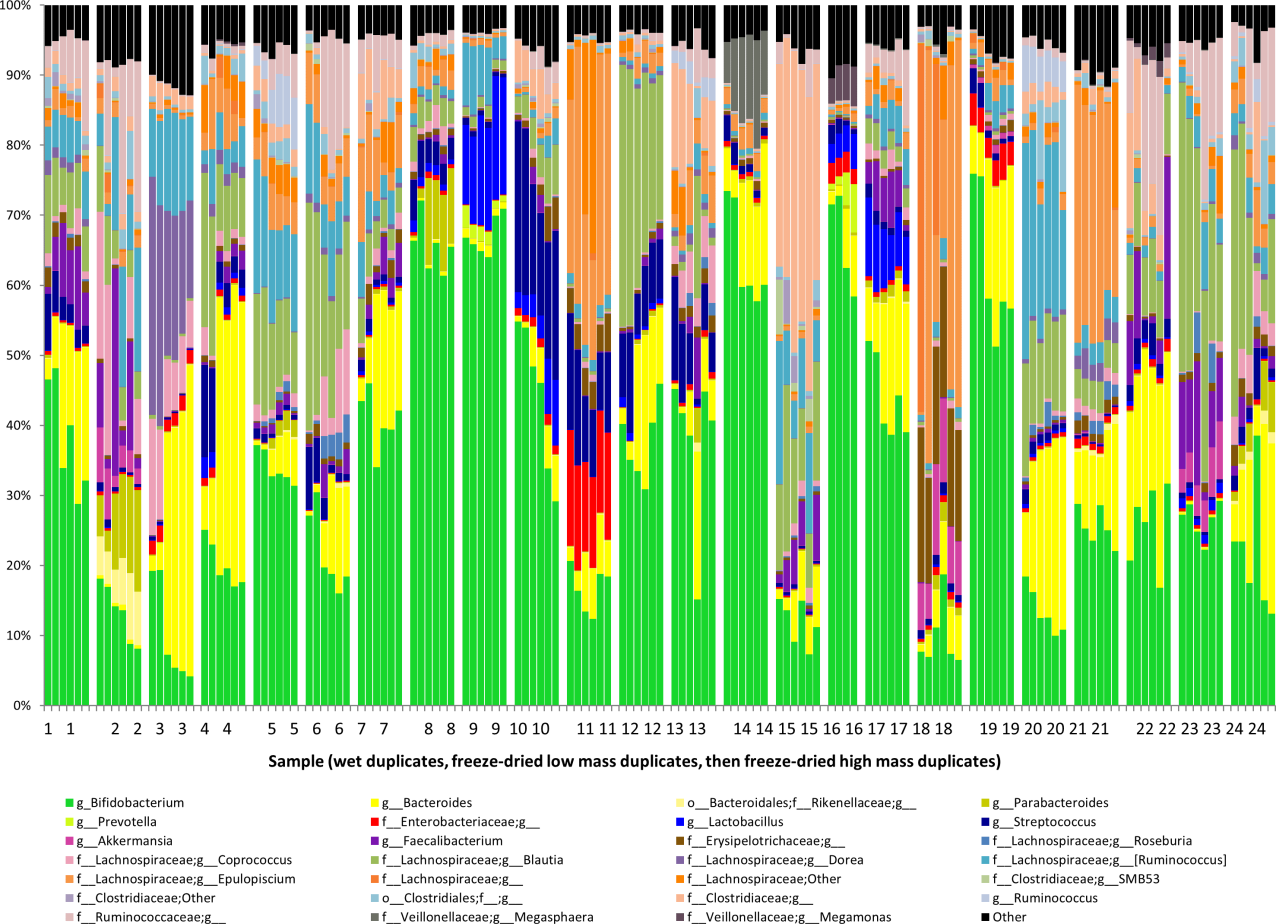
Bacterial communities of freeze-dried and wet feces. The bacterial community structures of the feces of 24 test infants. Relative abundances of each bacterial taxon are shown. Each sample is grouped together, with the two wet replicates first, followed by the two low-mass duplicates, and lastly the high-mass duplicates.

## Results

### Sample processing and summary description

After freeze-drying, the 24 fecal samples weighed on average approximately 25% of their pre-lyophilization weight, with a range of 14–32% (Table S1). Sequencing of the 16S ribosomal marker gene showed that the average fecal microbiota of these subjects was dominated by *Bifidobacterium*, followed in abundance by *Bacteroides*, and then by various members of the family Lachnospiraceae (Fig. 1). The average total fecal oligosaccharide level was 4.5 × 10^6^ ion counts (for an injection concentration of 1 mg/100 µL). The average relative abundance of fecal oligosaccharides types were 20.9% fucosylated (no sialic acid), 4.1% sialylated (no fucose), 2.9% fucosylated and sialylated, and 72.0% non-fucosylated neutral. These relative abundances for each glycan class are similar to previous results, with slightly lower relative sialylation and higher non-fucosylated neutrals (De Leoz et al., 2013).

### Freeze-drying effect on oligosaccharide profile

**Figure 2:**
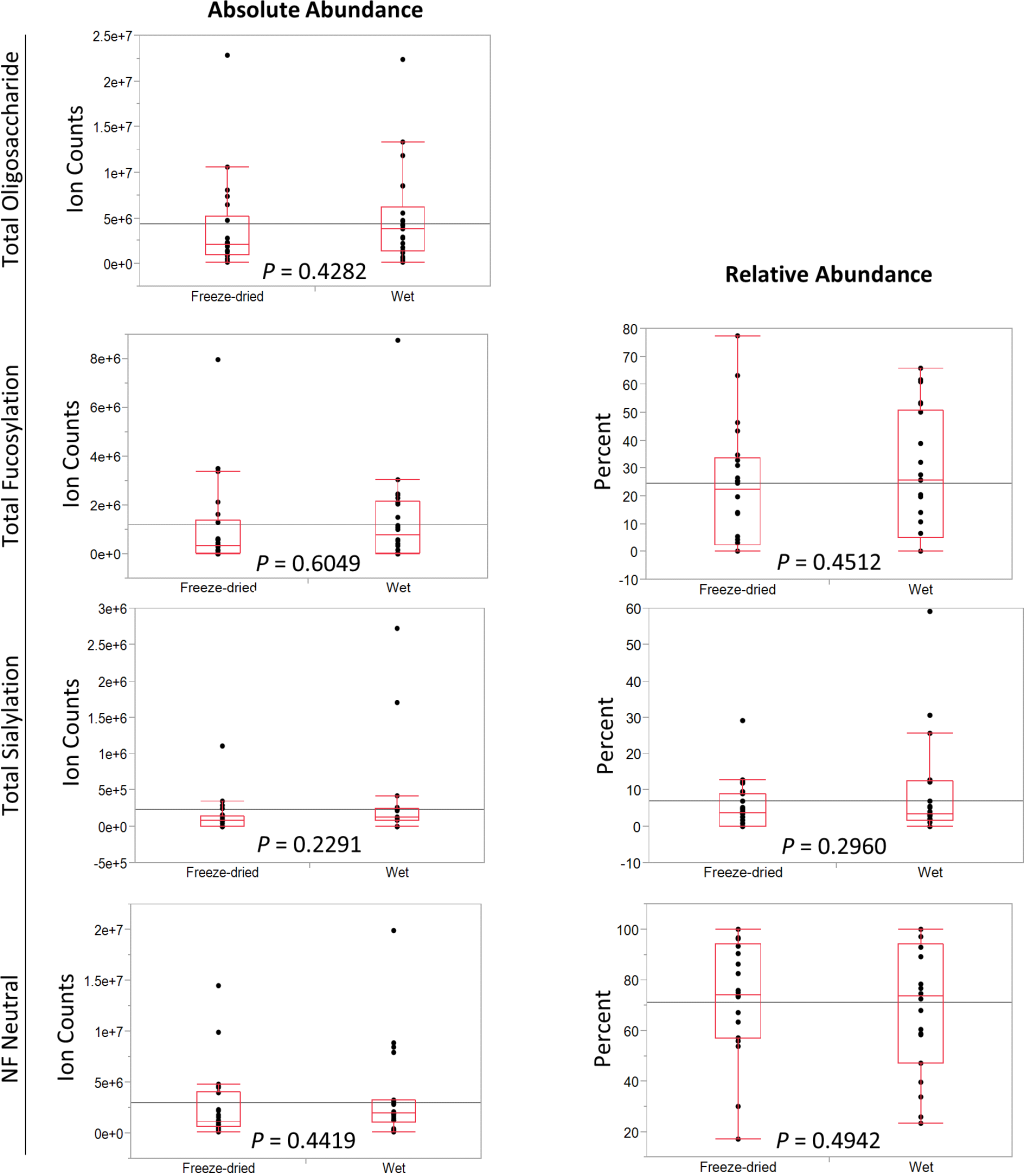
Comparison of fecal oligosaccharide measures between freeze-dried and wet fecal samples. Absolute and relative abundances of fecal oligosaccharides.

To test whether or not freeze-drying feces affects the oligosaccharide extraction and analysis, student’s *t*-tests were used to compare the extracted glycans. The absolute and relative abundances were compared between the two groups. Freeze-drying had no effect on the analysis of oligosaccharides, for both absolute and relative measures of oligosaccharides (Fig. 2). There was no significant difference in total (*p* = 0.4574), fucosylated and sialylated (*p* = 0.2552), fucosylated (*p* = 0.6084), sialylated (*p* = 0.2153), or non-fucosylated neutral oligosaccharides (*p* = 0.4985). There was also no significant difference for relative abundances between the two groups.

### Freeze-drying treatment effect on microbiome

**Figure 3:**
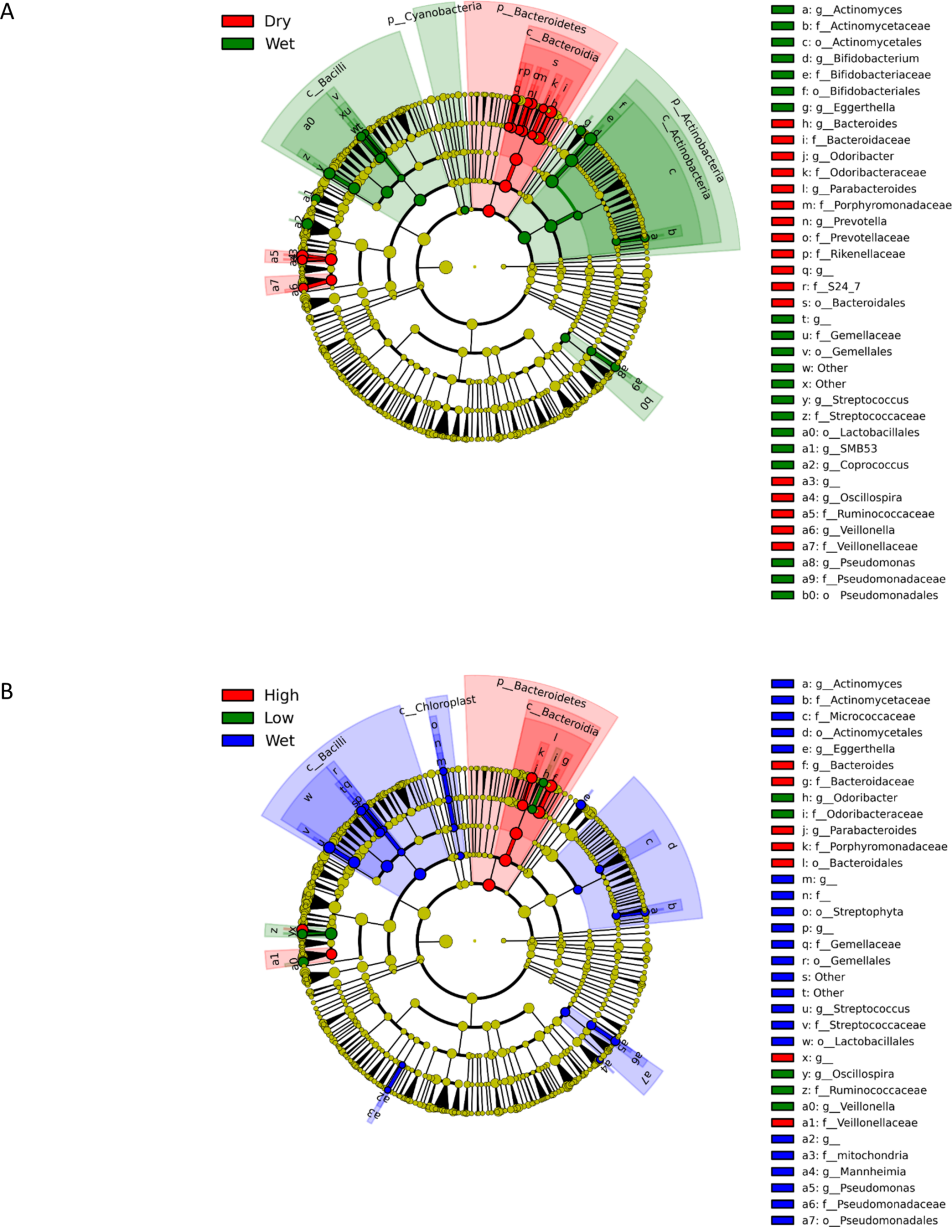
Cladograms representing the taxa enriched under various treatments. (A) shows the differential features of wet and dry (both high- and low-mass) replicates in an all-against-all comparison (classes = wet and dry; subclasses = wet, high, and low). (B) shows the results separated by high-mass and low-mass as well (classes = wet, high, and low) in a one-against-all comparison.

To test whether freeze-drying had any effect on measures of the fecal microbial community, the sequencing data was analyzed using Linear Discriminant Analysis Effect Size (LefSe) with default settings (unless otherwise noted) (Segata et al., 2011). Figure 3A shows the differential features of wet and dry (both high- and low-mass) replicates in an all-against-all comparison (classes = wet and dry, subclasses = wet, high, and low). The largest bacterial groups different between the two treatments were the class Bacilli, phylum Actinobacteria (higher in wet feces), and the phylum Bacteroides (higher in dried feces). Figure 3B shows the results separated by high-mass and low-mass as well (classes = wet, high, and low) in a one-against-all comparison, which produced a similar result. LefSe found no discriminative features in an all-against-all comparison between high-mass, low-mass, and wet classes, suggesting that high- and low-mass replicates were not significantly different. Figure S1 gives the Linear Discriminate Analysis scores for the differences listed in Fig. 3 (cutoff at 2.0).

**Figure 4:**
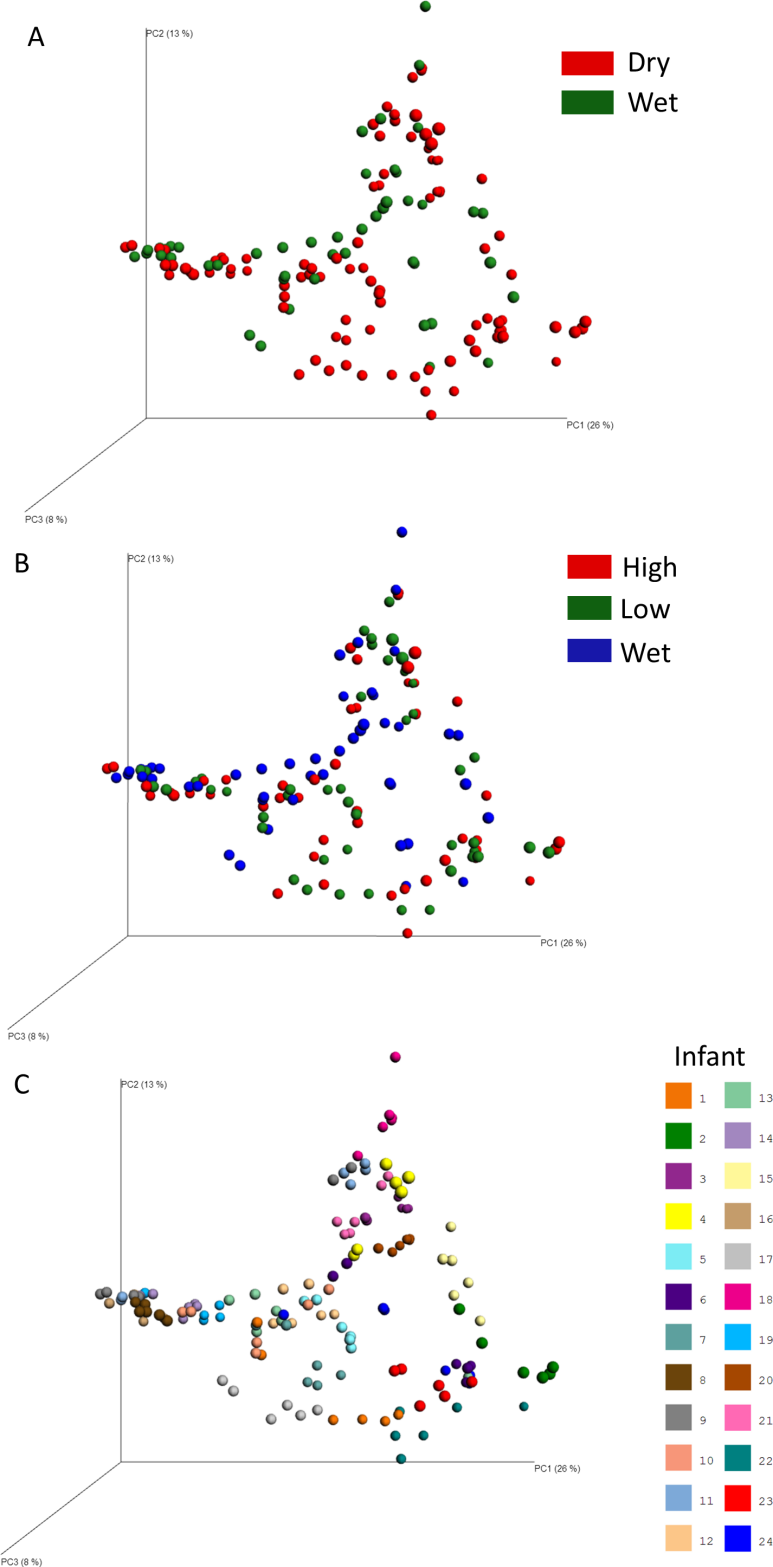
Principle Coordinates Analysis (PCoA) plots showing the clustering of samples by various metadata. (A) by Wet vs. Dry, (B) Wet vs. High Mass Lyophilized vs. Low Mass Lyophilized, (C) by Infant (subject).

To test the overall similarity of measures of the microbial communities from freeze-dried and wet feces, a weighted UniFrac distance matrix was calculated for all samples and tested statistically using the ANOSIM (Analysis Of Similarity) algorithm (Clarke, 1993; Lozupone & Knight, 2005; Fierer et al., 2010) implemented within QIIME. Three different groupings were tested: (1) wet vs. dry (both types), (2) wet vs. high mass lyophilized vs. low mass lyophilized, and (3) grouping by infant (test subject) shown in Table 1. There was no statistical support for grouping by wet vs. dry (*R* statistic = 0.008, *p*-value = 0.358), which suggests that lyophilization is not driving differences in the samples. Wet vs. high mass vs. low mass grouping had a significant *p*-value of 0.005, however the *R* statistic was very low (0.035), indicating the magnitude of the influence, though reproducible, was small. As expected, microbiota grouping by infant (subject) was significant and robust (*p* = 0.001, *R* = 0.838). Figure 4 shows PCoA plots colored by each of the tested groupings, for visualization of the grouping.

**Table 1:** Analysis Of Similarity (ANOSIM) analysis by various groupings. *P* values represent statistical significance, *R* statistics show effect size.

Classes	Method name	*R* statistic	*p*-value	Number of permutations
Wet vs. Dry	ANOSIM	0.008	0.358	999
Wet vs. High vs. Low	ANOSIM	0.0351	0.005	999
Infant (subject)	ANOSIM	0.8386	0.001	999

## Discussion

Research on the dramatic changes that occur in the gastrointestinal tract of neonates is increasing and the influence of diet (breast milk, formula and weaning foods) on the gastrointestinal tract (GIT) microbiota and its function are a particular focus (Zivkovic et al., 2013). We have previously noted correlations between the milk oligosaccharide content of the nursing mother and infant fecal microbiota composition (Lewis et al., 2015). Additional research has identified intriguing correlations between the milk oligosaccharide content remaining in the neonate feces and the fecal microbiota composition (De Leoz et al. (2014); Wang et al. (2015)) clearly suggesting specific primary consumers (i.e., bifidobacteria) among the early colonizers of the infant GIT. However more studies comparing fecal glycome and microbiome are needed, particular in infants at risk for malnutrition (Subramanian et al., 2015) in developing countries.

Lyophilization is a common preservation and mass-reduction method for fecal samples obtained from remote field sites or stored at local collection points without the need for a cold chain transportation or consistent electrical power to on-site freezers. The use of this method could expand and diversify the set of possible sampling locations, however, the effects of freeze-drying on measures of microbial communities, their determinants (e.g., human milk oligosaccharides), and their downstream byproducts must first be understood. As the fecal oligosaccharide profile is essential to a comprehensive understanding of the nursing infant gut ecosystem due to the selective pressures it exerts, the impact of freeze-drying on the milk-derived oligosaccharide profile of infant fecal samples was tested. Encouragingly, the data showed oligosaccharide profiles were not influenced by the lyophilization process.

As marker gene amplicon sequencing is the *méthode de jour* for studying microbial ecology in a high-throughput manner, we applied the method to matched freeze-dried and control samples. The goal was to investigate whether freeze drying produced any systematic bias in the detected relative abundance of different bacterial taxa. As expected for mostly breast-fed infants of approximately 1 year of age, the bacterial communities in these infants was dominated by bifidobacteria and *Bacteriodes*, with an appreciable presence of Lachnospiraceae (Fallani et al., 2011; Azad et al., 2013; Bergström et al., 2014). While the ANOSIM results showed that the variation in community structure between conditions was much lower than the variation between infants, there were some differences between freeze-dried and wet feces. We found differences in the two treatments, consistent with Ruiz et al., who found increases in RFLP (Restriction Fragment Length Polymorphism) band brightness in some bands after lyophilizing feces, suggesting an increase in the recovery of DNA from some species, and not others (Ruiz & Rubio, 2009).

At first glance, differences in cell wall structure appear to be a possible driver of the differences in our data. The Gram-negative *Bacteroides* was over-represented in lyophilized feces, while Gram-positive bifidobacteria and bacilli were comparatively under-represented. Measured *Bacteroides* and bifidobacterial abundances have previously been shown to be sensitive to variation in extraction method (Milani et al., 2013; Wesolowska-Andersen et al., 2014). As our data is based on relative abundances, the increase in the detection of one species would be read as a concomitant decrease in the abundances of all other species. However, the inclusion of the spore-forming Gram-positive *Oscillospira* with taxa more abundant in lyophilized feces and the Gram-negative *Pseudomonas* with taxa more abundant in wet feces casts doubt on cell morphology as the main driver of detection differences between conditions. It should be noted, however, that the taxomonic level at which these two taxa were discriminative was narrower than the previously mentioned groups, suggesting the existence of genus-level (or lower) explanations for the effects of lyophilization on these taxa. Notably, *Oscillospira* and *Pseudomonas* were also minor members of the community. Mechanistic explanations on the cause of these minor differences observed between freeze-dried and non-freeze-dried samples remain elusive and warrant further study. Regardless of mechanism, such differences must be considered when performing studies of microbial communities from lyophilized feces.

We also investigated the unstudied issue of the appropriate amount of lyophilized sample to load into commercial DNA extraction kits. Manufacturer’s instructions often direct that a set mass (or range of masses) of sample to be used, but to the best of our knowledge, no manufacturer tests or validates their kit on lyophilized fecal samples. The appropriate amount of sample to use is unclear, as (for example) 100 mg of fresh feces is not equal to 100 mg of freeze-dried feces, due to the concentration of other sample constituents induced by water loss during the freeze-drying process. Due to concerns about overloading the ZR Fecal DNA Miniprep kits with a concentrated freeze-dried sample (either biasing the lysis of cells or overwhelming the binding capacity of the DNA purification column) we also tested a mass correction procedure. The data also showed that there were no appreciable differences in the relative abundances of taxa detected from DNA extracted from the high-mass (no correction for concentration of feces during freeze-drying) and low-mass (correction applied) lyophilized samples. This data provides support for the use of lower amounts of lyophilized feces in similar future studies. As we encountered difficulty fully re-hydrating several of the high-mass samples with the default amount of lysis buffer, it is recommended to use mass-adjusted freeze-dried samples for that reason alone.

Although this study contributes knowledge on the effect of lyophilization on marker gene sequencing and oligosaccharide profiles, a few caveats must be noted. First, this study was performed with infant fecal samples and with a single method each of DNA and oligosaccharide extraction. Studies using other types of biological samples or other oligosaccharide and DNA extraction methods may or may not develop the same results. Second, there is no reason to think that the measurements from wet feces are more accurate than those from freeze-dried feces. It is unknown whether the DNA obtained after lyophilization might be more representative of the community than that from wet DNA. If the extraction efficiency of previously under-represented species is increased, the profiles from lyophilized feces might be preferred.

## Conclusion

While some differences in the microbial community measures of freeze-dried and wet feces were apparent, the effect size was small, and differences between absolute and relative oligosaccharide abundances were not significant. Individual microbial community variation between samples was still readily apparent from lyophilized fecal matter. The decision to utilize freeze-dried samples or not is hypothesis-dependent and must be made with caution. Comparing within a sample set of all freeze-dried samples would eliminate any treatment effects of lyophilization on measures of microbial communities, however further study is needed to identify the mechanism of bias introduction and thus predict the impact of this sample treatment. Comparisons of microbial communities where one sample set was freeze-dried and the other fresh, must acknowledge the differences introduced by sample processing which could introduce error and bias conclusions. The ZR Fecal DNA Miniprep kit used in this study appears to maintain unbiased DNA extraction under the increased inputs of freeze-dried samples where the concentration of samples is not accounted for. However, due to the limited sample quantity available in many studies, we imagine most researchers will wish to reduce sample mass used for DNA extraction to the equivalent amount of wet sample, as the results appear nearly identical.

In summary, our results show that lyophilization is unlikely to obscure major differences in microbial community structure and oligosaccharide content between classes of samples and thus is an acceptable method of sample preservation for the purposes of studying microbial communities and milk oligosaccharide profiles in infants from remote locations. However, its limitations must still be considered when drawing conclusions. Numerous different fields of research are recognizing the importance of interrogating diverse ecosystems and environments and people that are not as easily accessible to the researchers in Western, Educated, Industrialized, Rich, and Democratic (“WEIRD”) countries (Henrich, Heine & Norenzayan, 2010). Additional exploration of other potential sample preservation methods will further inform best practices for sample integrity; however, consideration must be given to the practicalities of sample collection and preservation in areas with limited access to the funds and material necessary to implement these best practices. The techniques described in this study can aid in diversifying the sample sets and cohorts of infants from which samples may be obtained due to the minimal maintenance needed by freeze-dried samples. The additional perspective gained from the study of infants from different environmental contexts is key to understanding the early stages of human co-development with our microbiota.

## Supplemental Information

10.7717/peerj.1612/supp-1 Table S1 Weights before and after freeze-drying for each sample Contains Metadata for each sample.

10.7717/peerj.1612/supp-2 Figure S1 LDA (Linear Discriminate Analysis) Scores for cladograms in Fig. 2
LDA cutoff for significance was set at 2.0.

## Additional Information and Declarations

### Data Availability

The following information was supplied regarding data availability: The sequencing data is available in the European Nucleotide Archive under study number ERP012928.

### Clinical Trial Registration

The following information was supplied regarding Clinical Trial registration: ClinicalTrials.gov Identifier: NCT01817127. No CONSORT flowchart needed as this is an observational trial, not an interventional one.
