# Supplementary material for: The impact of freeze-drying infant fecal samples on measures of their bacterial community profiles and milk-derived oligosaccharide content
Source: PeerJ. 2016 Jan 21;4:e1612. doi: 10.7717/peerj.1612 (PMC4727960; doi:10.7717/peerj.1612)
Supplement: Table S1 — Contains Metadata for each sample. [file peerj-04-1612-s001.pdf]

Supplementary Table 1

Infant (Subject) number	UC Davis Lactation Study ID	Day of Life	Received Breast Milk	Wet feces mass (split between two duplicates) (mg)	Sample mass before freeze drying (g)	Sample mass after freeze-drying (g)	Percent of original weight	Freeze-dried mass (split between two duplicates) (mg)
1	1038	345	Unknown	320	1.7	0.498	29.29412	87.88235
2	1037	345	No	259	1.68	0.245	14.58333	43.75
3	1041	345	Yes	278.3	1.3	0.336	25.84615	77.53846
4	1036	345	Unknown	297.4	1.74	0.398	22.87356	68.62069
5	1034	349	No	359.8	1.7	0.51	30	90
6	1039	345	No	338	1.6	0.387	24.1875	72.5625
7	1007	345	No	357.9	1.77	0.544	30.73446	92.20339
8	1005	345	Yes	350.8	1.73	0.562	32.48555	97.45665
9	1002	345	Unknown	335.5	1.73	0.46	26.5896	79.76879
10	1004	344	Yes	311.8	1.73	0.341	19.71098	59.13295
11	1003	345	Yes	200.8	1.54	0.279	18.11688	54.35065
12	1001	346	No	367	1.71	0.445	26.02339	78.07018
13	1028	345	No	313	1.7	0.475	27.94118	83.82353
14	1019	345	Yes	340	1.71	0.315	18.42105	55.26316
15	1014	345	Yes	307.8	1.8	0.337	18.72222	56.16667
16	1012	345	Yes	140.8	0.92	0.254	27.6087	82.82609
17	1011	345	Yes	320.8	1.8	0.509	28.27778	84.83333
18	1010	393	Yes	348	1.77	0.44	24.85876	74.57627
19	1040	351	No	320	1.75	0.383	21.88571	65.65714
20	1032	346	Yes	102.8	1.62	0.268	16.54321	49.62963
21	1031	346	Yes	277.3	1.7	0.497	29.23529	87.70588
22	1015	345	Yes	298.2	1.71	0.317	18.53801	55.61404
23	1023	345	Yes	286	1.74	0.49	28.16092	84.48276
24	1029	345	Yes	333	1.85	0.425	22.97297	68.91892
Average							24.31714	72.95142
