# Supplementary figures and images for: The impact of freeze-drying infant fecal samples on measures of their bacterial community profiles and milk-derived oligosaccharide content
Source: PeerJ. 2016 Jan 21;4:e1612. doi: 10.7717/peerj.1612 (PMC4727960; doi:10.7717/peerj.1612)

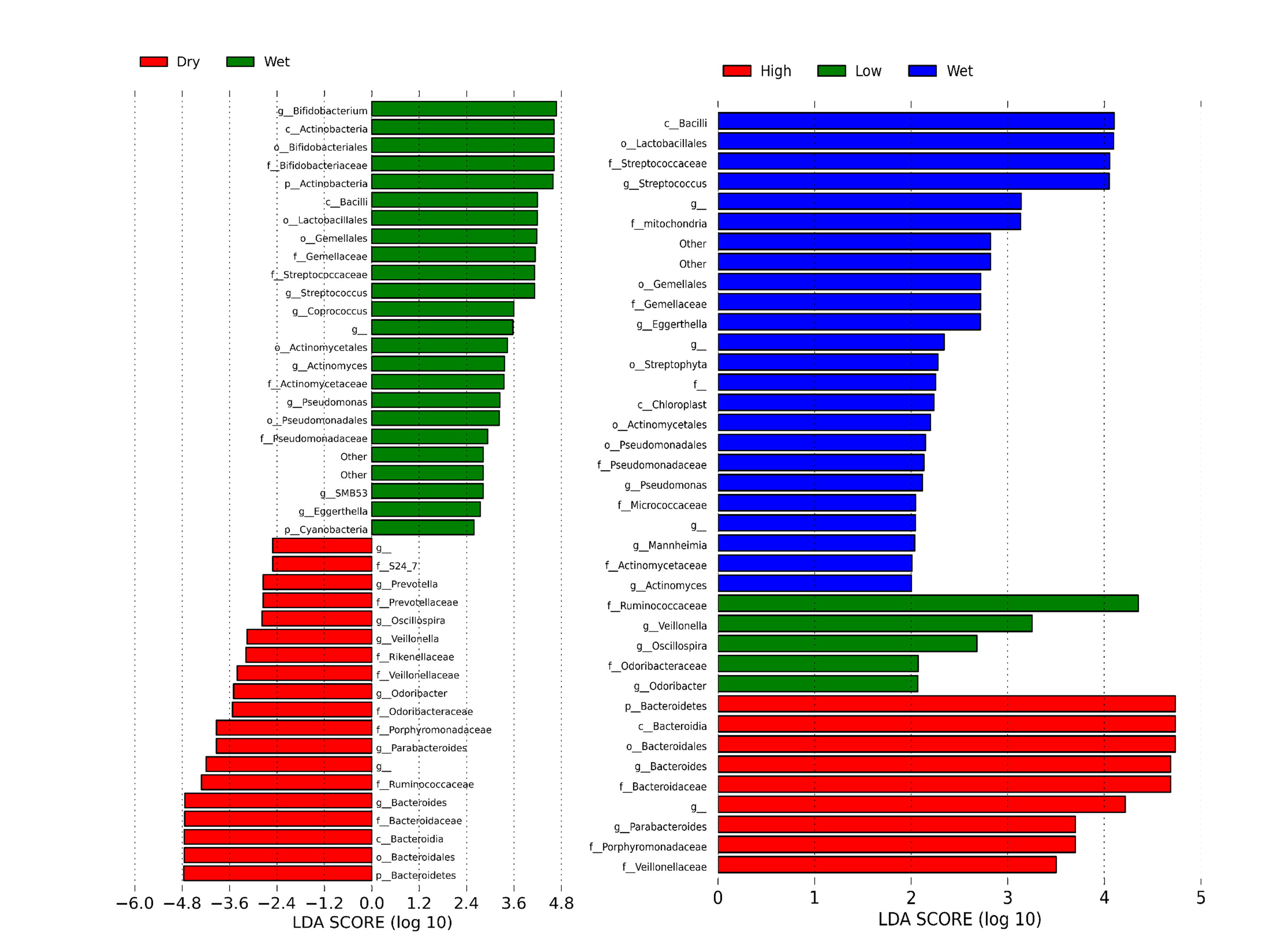

Supplement: Figure S1 — LDA cutoff for significance was set at 2.0. [file peerj-04-1612-s002.png]
